# Supplementary material for: A study of bacteria adhesion and microbial corrosion on different stainless steels in environment containing Desulfovibrio vulgaris
Source: R Soc Open Sci. 2021 Jan 13;8(1):201577. doi: 10.1098/rsos.201577 (PMC7890485; doi:10.1098/rsos.201577)
Supplement: Figs. S1 and S2 [file rsos201577supp1.docx]

Figure S1 Correlation between cells adhesion (assay 1) and Hydrophobicity

Figure S2 Correlation between cells adhesion (assay 2) and corrosion rate
